# Rabies Postexposure Prophylaxis, New York, 1995–2000

**DOI:** 10.3201/eid1112.041278

**Published:** 2005-12

**Authors:** Jesse D. Blanton, Nadine Y. Bowden, Millicent Eidson, Jeffrey D. Wyatt, Cathleen A. Hanlon

**Affiliations:** * Centers for Disease Control and Prevention, Atlanta, Georgia, USA; † New York State Department of Health, Albany, New York, USA; ‡ University of Rochester School of Medicine & Dentistry, Rochester, New York, USA

**Keywords:** Rabies, epidemiology, animal exposure, vaccination, zoonoses, research

## Abstract

The epidemiology of human rabies postexposure prophylaxis (PEP) in 4 upstate New York counties was described from data obtained from 2,216 incidences of PEP recorded by local health departments from 1995 to 2000. Overall annual incidence for the study period was 27 cases per 100,000 persons. Mean annual PEP incidence rates were highest in rural counties and during the summer months. PEP incidence was highest among patients 5–9 and 30–34 years of age. Bites accounted for most PEP (51%) and were primarily associated with cats and dogs. Bats accounted for 30% of exposures, more than any other group of animals; consequently, bats have replaced raccoons as the leading rabies exposure source to humans in this area.

## Abstract 2

Bats are now the leading source of rabies postexposure prophylaxis.

Combined with effective human rabies prophylaxis, canine rabies control programs were responsible for the steady decline of human rabies in the United States, from 20–25 annual cases in the 1940s to <3 annual cases in the 1990s (*1**–**4*). Although the current incidence of human rabies in the United States is negligible compared to that of other infectious diseases, the number of persons seeking rabies postexposure prophylaxis (PEP) is high; 18,238 persons received PEP in New York (excluding New York City) from 1993 to 1998 (*5*). No proven curative treatment has been documented for rabies once clinical disease begins (*6*). Human rabies can be prevented by following the Advisory Committee on Immunization Practices (ACIP) recommendations of local wound care and prompt administration of human rabies immune globulin (HRIG, 20 IU/kg) on day 0 and vaccine on days 0, 3, 7, 14, and 28 (*7*). For persons who have been previously vaccinated, the recommended prophylaxis consists of a vaccine dose on days 0 and 3.

Studies addressing rabies PEP incidence indicate a rising trend since the 1970s. Estimates of annual PEP incidence in Georgia increased from 1.94 cases/100,000 in 1970 to an estimated 6.17 cases/100,000 from 1995 to 2001 (1, S.J. Onufrak, Source-specific risks among patients receiving rabies post-exposure prophylaxis in Georgia [master's thesis]. Atlanta: Emory University; 2003). At the national level, incidence was most recently estimated at 8.69 cases/100,000 in 1980 (*8*). Increases are probably attributable to an expanding raccoon rabies epizootic in the mid-Atlantic states and changes in PEP consideration after potential bat exposure (*5*). We describe demographic and animal exposure data associated with PEP in upstate New York several years after the establishment of the raccoon rabies variant and compare them with 1993–1994 data from the same area (*9*).

## Methods

Monroe and Onondaga Counties encompass the cities of Rochester and Syracuse and are predominantly urban-suburban with population densities of 422 and 232 persons/km^2^, respectively. Cayuga and Wayne Counties are predominantly rural-suburban, with population densities of 46 and 60 persons/km^2^, respectively. The 4-county region in western upstate New York is 7,086 km^2^, with an estimated human population of 1,369,407 (*10*).

We considered all PEP cases recorded on standardized reports by the 4 local health departments from 1995 to 2000. Data included patient demographics, animal characteristics, and exposure details. The report form was changed in 1998, with the addition of age, sex, treatment dates, and more detailed exposure information for bat-related PEP. Age and sex data were obtained directly from local health departments for PEP cases before 1998.

Exposure source was defined as the suspected or confirmed rabid animal that directly or indirectly resulted in potential human exposure. Direct exposure consisted of a bite, scratch, or contamination of mucous membrane with potentially infectious material directly from a suspected rabid animal. Indirect exposure consisted of contact with potentially contaminated fomites (e.g., saliva from a pet's fur that comes into contact with open wounds or mucous membranes). Cases that lacked specific information about route of exposure were classified as unspecified. Cryptic or unspecified bat exposures consisted of discovering a bat in a room with a sleeping person, unattended child, mentally impaired person, intoxicated person, or someone otherwise unable to rule out contact. Rabies diagnostic results were obtained on animal cases from the New York State Department of Health Wadsworth Center Rabies Laboratory. Population data from the 2000 US census were used to calculate the incidence of PEP by county, age, and sex (*10*). Statistical analyses, including frequencies and chi-square tests, were performed with the SAS statistical package version 8.0 (SAS Institute Inc., Cary, NC, USA).

## Results

A total of 2,216 PEP cases were reported from the study area from 1995 to 2000, with 317–469 cases each year. Annual PEP incidence was 23–34 cases/100,000 during the 6-year period (average 27/100,000). The mean annual incidence for the urban counties of Monroe and Onondaga (319 residents/km^2^) was 23 cases/100,000 compared to 56 cases/100,000 in the rural counties of Cayuga and Wayne (52 residents/km^2^). No failures of PEP were recorded.

**Figure 1:**
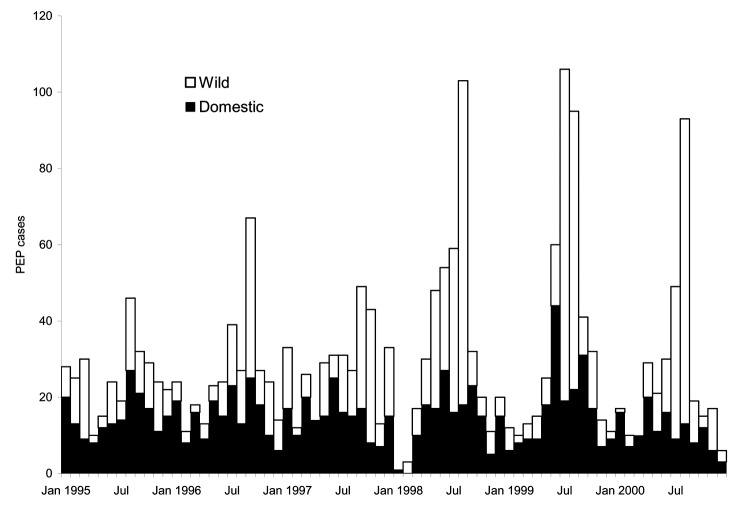
Human rabies postexposure prophylaxis (PEP) by month and species of exposure (domestic vs. wild), 4 upstate New York counties (Cayuga, Monroe, Onondaga, and Wayne), 1995–2000.

**Figure 2:**
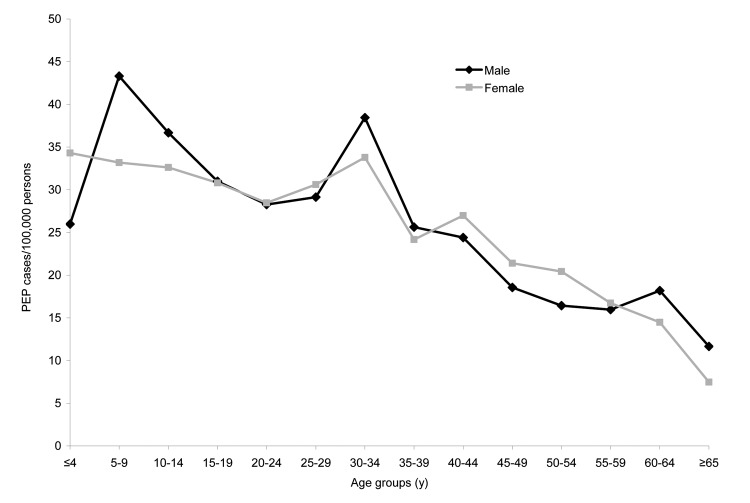
Human rabies postexposure prophylaxis (PEP) incidence by sex and age group, 4 upstate New York counties (Cayuga, Monroe, Onondaga, and Wayne), 1995–2000.

PEP cases tended to increase in the late spring/early summer; the highest number of PEP cases was seen in August/September in 1996 and 1997 and in July/August from 1998 to 2000 (Figure 1). Of 2,109 (95%) PEP cases for which sex data were available, 51% were male. The median age of PEP recipients was 27 years for men and 29 years for women. The mean annual incidence of PEP for men was 26 cases/100,000 and for women 24 cases/100,000. PEP incidence rates were highest in persons 5–9 years of age, followed by those 30–34 years of age (Figure 2). No significant differences among sex or age distributions and PEP were seen.

Wild animal exposures accounted for 1,081 PEP cases (49%), domestic animals accounted for 1,057 cases (48%), and species of exposure animal was not identified for 78 cases (3%) (Table 1). Bats accounted for 663 (61%) PEP cases related to wildlife exposures, while other sources of wildlife-related PEP included raccoons (250 cases), foxes (85 cases), skunks (46 cases), woodchucks (12 cases), opossums (6 cases), deer (5 cases), beavers, coyotes, and squirrels (3 cases each), and other wild species (5 cases). PEP from bat exposure was significantly associated with an urban setting (p<0.001). Among domestic animal exposures that resulted in PEP, 523 were attributed to cats, 498 to dogs, 19 to cattle, 11 to horses, 4 to ferrets, and 1 each to a pet rabbit and monkey.

**Table 1:** Human rabies postexposure prophylaxis (PEP) by animal source, 4 counties, New York, 1995–2000*

Animal source	Bite, n (%)	Nonbite, n (%)			Unspecified§	Total, n (%)
		Direct		Indirect‡		
		Scratch	Saliva/NT†			
Raccoon	48 (19)	16 (6)	65 (26)	120 (48)	1 (<1)	250 (11)
Bat (all species)	115 (17)	29 (4)	100 (15)	11 (2)	408 (62)	663 (30)
Other wild species¶	76 (45)	6 (4)	41 (24)	44 (26)	1 (1)	168 (8)
All wild species	239 (22)	51 (5)	206 (19)	175 (16)	410 (38)	1,081 (49)
Cat	367 (70)	64 (12)	89 (17)	3 (1)	0	523 (24)
Dog	493 (99)	0	3 (1)	0	2 (<1)	498 (22)
Other domestic species#	7 (19)	0	28 (78)	0	1 (3)	36 (2)
All domestic species	867 (82)	64 (6)	120 (11)	3 (<1)	3 (<1)	1,057 (48)
Unknown	22 (28)	4 (5)	19 (24)	28 (36)	5 (7)	78 (3)
Total	1,128 (51)	119 (5)	345 (16)	206 (9)	418 (19)	2,216 (100)

*Data are from Cayuga, Monroe, Onondaga, and Wayne Counties.

†Direct contamination of an open wound or mucous membrane with potentially infectious material such as saliva or neural tissue (NT).

‡No known direct contact with a rabid or suspected rabid animal. Indirect exposure consisted of possible contact with saliva on an animal (i.e., pet dog or cat) or inanimate object from a suspected rabid animal that resulted in contamination of an open wound or mucous membrane.

§Unspecified contact indicates no exposure information was listed or exposure was indicated as unknown on data records. Unspecified exposure for bats includes being in the physical presence of a bat and not being able to rule out direct contact, particularly a bite. More people received PEP after unspecified exposure to bats than any other group of animals (p<0.001).

¶Includes beaver, coyote, chipmunk, deer, fox, mouse, opossum, otter, rat, skunk, squirrel, and woodchuck.

#Includes cow, ferret, horse, monkey, and rabbit.

Animals were not available for observation or testing for 66% of rabies PEP cases that resulted from exposure to cats and 89% that resulted from exposures to dogs. Of the dog-associated PEP, significantly (p<0.001) more of them (93%) occurred in urban counties compared to rural counties (Table 2). During the study period, only 16 (3%) dog-associated PEP cases involved dogs that were tested for rabies, and none were confirmed rabid. Among cats, 132 (25%) cat-associated PEP cases involved cats that were tested for rabies; of these, 110 PEP cases (83%) involved exposure to a confirmed rabid cat.

**Table 2:** Human rabies postexposure prophylaxis (PEP) by setting, 4 counties, New York, 1995–2000*

Animal source	Urban, n (%)	Rural, n (%)
Dog†	463 (93)	35 (7)
Cat	386 (74)	137 (26)
Other domestic‡	16 (44)	20 (56)
All domestic	865 (82)	192 (18)
Raccoon	162 (65)	88 (35)
Bat§	456 (69)	207 (31)
Fox	50 (59)	35 (41)
Skunk	28 (61)	18 (39)
Other wild¶	19 (51)	18 (49)
All wild	715 (66)	366 (34)
Total#	1,580 (74)	558 (26)
Annual rate/100,000	22.6	56.9

*Rabies PEP cases reported to the health departments of 2 relatively urban counties, Onondaga and Monroe, and 2 relatively rural counties, Cayuga and Wayne.

†Human PEP cases from dog exposures were significantly higher in urban counties (p<0.001).

‡Other domestic animal exposures included 2 cows (10 cases), 3 ferrets (4 cases), 1 monkey (1 case), and 1 rabbit (1 case) in urban counties and 5 cows (9 cases) and 2 horses (11 cases) in rural counties.

§Human PEP cases due to bat exposures were significantly higher in urban counties (p<0.001).

¶Other wild animal exposures included 8 woodchucks (8 cases), 4 opossums (5 cases), 1 beaver (2 cases), 1 rat (1 case), 1 coyote (1 case), 1 mouse (1 case), and 1 otter (1 case) in urban counties and 5 deer (5 cases), 4 woodchucks (4 cases), 2 squirrels (3 cases), 2 coyotes (2 cases), 2 chipmunks (2 cases), 1 beaver (1 case), and 1 opossum (1 case) in rural counties.

#78 PEP cases excluded because animal source was missing.

A total of 1,128 (51%) PEP cases were attributed to animal bite; 670 (30%) persons reported nonbite exposures, and 418 (19%) reported exposure as unknown or unspecified (Table 1). Among nonbite-associated PEP recipients, 69% reported direct animal contact. Of the 1,106 bite-related PEP recipients that reported the species, 78% involved domestic animals. In 62% of potential exposures to bats, an exposure route was not described.

Exposure of only 1 person to a suspected rabid animal precipitated 1,336 (60%) PEP cases (Table 3). Exposure of a single person was more likely to be associated with a bite (p<0.001). Wild animal species accounted for 72% of group exposure PEP. The largest group occurred in June 1999, when 29 persons received PEP after exposure to a rabid cat.

**Table 3:** Human rabies postexposure prophylaxis (PEP) by group size, 4 counties, New York, 1995–2000

Characteristic	Group size, n (%)					
	1	2	3	4	5	>6
No.	1,336 (60)	284 (13)	159 (7)	192 (9)	55 (2)	190 (9)
No. sources	1,336 (83)	142 (9)	53 (3)	48 (3)	11 (1)	18 (1)
Route of exposure*						
Bite†	1,008 (75)	69 (24)	18 (11)	6 (3)	1 (2)	26 (14)
Nonbite	316 (24)	205 (72)	132 (83)	170 (89)	49 (89)	163 (86)
Unknown	12 (1)	10 (4)	9 (6)	16 (8)	5 (9)	1 (<1)
Source of exposure*						
Dog or cat	845 (63)	50 (18)	27 (17)	4 (2)	5 (9)	90 (47)
Other domestic species	5 (<1)	12 (4)	0	4 (2)	0	15 (8)
Raccoon	111 (8)	44 (16)	21 (13)	32 (17)	2 (4)	40 (21)
Bat	241 (18)	132 (46)	96 (60)	147 (76)	43 (78)	4 (2)
Other wild species	97 (7)	36 (13)	9 (6)	5 (3)	0	21 (11)
Unknown source animal	37 (3)	10 (3)	6 (4)	0	5 (9)	20 (11)
Mean age (y)	30.9	31.6	23.8	22.5	16.2	26.6

*Route of exposure and source of exposure percentages calculated within group size to accommodate comparison.

†Bite exposure was significantly associated with single-person exposures vs. group exposures (p<0.001).

Laboratory diagnosis of rabies was sought in 249 animals associated with 515 PEP cases (23%). Contact with a wild animal accounted for 348 cases (68%) where laboratory diagnosis was sought. Raccoons accounted for 176 (57%) of 309 PEP cases attributed to confirmed rabid wildlife. Nonbite exposures accounted for 366 (73%) of 501 PEP cases in which a laboratory diagnosis of rabies was obtained. Laboratory diagnosis of rabies in the exposing animal was significantly associated with nonbite exposure (p<0.001).

From 1998 to 2000, the time of PEP initiation in relation to exposure was available for 1,219 (98%) of 1,248 cases. The period between exposure and treatment varied from 0 to 115 days with a median of 3 days. Among persons with bite exposure, 199 (38%) of 528 began PEP the same day as exposure, while 14% of persons who reported a nonbite exposure received treatment the same day as exposure (p<0.001). Medians of 1 day for wild animal exposures and 2 days for domestic animal exposures were associated with bite exposures and 3 and 6 days, respectively, for nonbite exposures.

Of 1,248 PEP cases reported from 1998 to 2000, administration of PEP biologics was recorded as complete and appropriate (e.g., HRIG was given if indicated and the person completed all 5 vaccinations) in 1,035 (83%) cases. A total of 62 persons (5%) had received prior vaccination; 47 (76%) completed the appropriate course of treatment. Among rabies vaccination–naive persons, 984 (85%) of 1,157 completed the appropriate course of treatment. Information regarding treatment scheduling was not available for 29 (2%) PEP cases.

Information on vaccine scheduling was available for 724 (58%) of the 1998–2000 PEP cases. Administration schedules were correct for 605 (84%) persons. Six persons (1%) did not receive HRIG when it was indicated, and 9 (1%) previously vaccinated persons received HRIG, although it was not indicated. One person received 6 total vaccine doses. Adverse events were listed as either present or absent with no scale as to severity. In all, 63 persons (5%) reported adverse reactions to vaccine or to HRIG.

## Discussion

Epidemiologic characteristics of possible rabies exposure leading to PEP changed substantially in this 4-county upstate New York area from 1993 to 2000. The major changes were the animal species exposure source and type of exposure (Table 4). From 1995 to 2000, overall PEP incidence declined in this area to 27 cases/100,000 from a high of 43 cases/100,000 in the early 1990s (*9*). Although affected by complex factors, this may reflect increased knowledge about what constitutes an exposure from terrestrial mammals among the public and healthcare providers and how to avoid exposures.

**Table 4:** Human rabies postexposure prophylaxis (PEP), 4 counties, New York, 1993–2000

Characteristic	1993–1994*	1995–2000
PEP cases (annual mean)	1,173 (587)	2,216 (369)
Annual PEP incidence	32/100,000 urban, 123/100,000 rural	23/100,000 urban, 57/100,000 rural
Season	Summer to early autumn	Summer to early autumn, July–August for 1998–2000
Sex	55% male (47/100,000), 45% female (38/100,000)	51% male (27/100,000), 49% female (25/100,000)
Age (y)†	10–14 and 35–55	5–9 and 30–34
Exposure source (%)		
Wild	67	51
Raccoon	50	12
Bat	5	31
Other	12	8
Domestic	33	49
Cat	17	24
Dog	14	23
Other	2	2
Exposure type (%)		
Bite	30	51
Scratch	6	5
Direct‡	14	16
Indirect§	51	28
Group size	47% >2 persons exposed	40% >2 persons exposed

*Data from Centers for Disease Control and Prevention (*9*).

†Age groups with highest annual incidence.

‡Direct exposure of saliva or neural tissue to wound or mucous membrane.

§Indirect exposure to saliva or neural tissue to wound or mucous membrane, includes unidentified exposures from 1995 to 2000.

In agreement with other recent studies, cats accounted for a majority of the exposures from domestic animals (*9*). Reinforced emphasis of responsible pet ownership and routine vaccination with specific attention to the ideal of maintaining cats indoors and up-to-date on their rabies vaccinations may help to reverse this trend. At this time, many states and localities do not require rabies vaccination in cats. New York established a statewide requirement for rabies vaccination for cats in 2002. Progress in this area would be further enhanced through tangible enforcement mechanisms.

The passive nature of PEP data collection is an inherent weakness in most studies addressing PEP incidence. The capture rate in this study is high because New York has a requirement for reporting all PEP cases and provided partial reimbursement to local health departments for uncovered expenses. However, some cases may not have been reported if costs were borne by the private sector.

**Figure 3:**
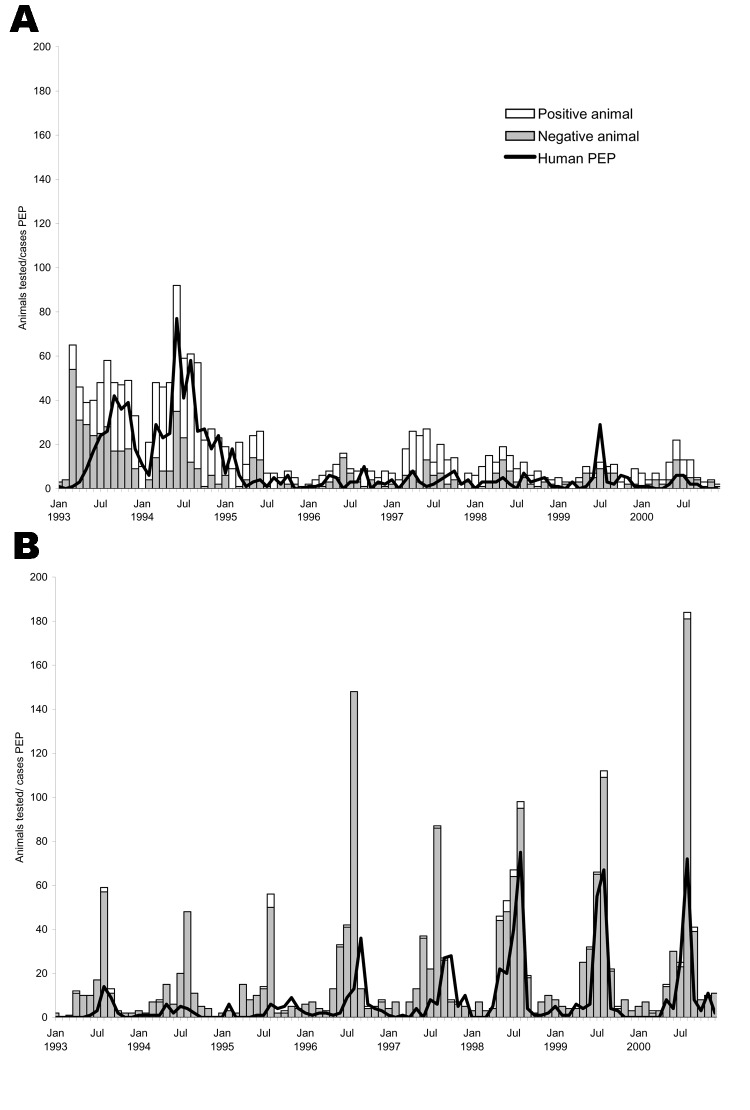
Human rabies postexposure prophylaxis (PEP) associated with raccoon (A) or bat (B) exposures and the number of raccoons or bats that tested positive or negative for rabies, 4 upstate New York counties (Cayuga, Monroe, Onondaga, and Wayne), 1993–2000.

Potential exposures to bats have replaced raccoons as the most common species leading to PEP (Figure 3). By 1998, bats had become the leading source of exposure for which PEP was sought in this area. Historically, bats have only accounted for 5% to 10% of PEP cases (*1**,**8**,**9*). Furthermore, most exposures to bats (62%) were cryptic or listed as unknown; in other words, the exposure could not be described as a bite from a bat or as direct or indirect contamination of an open wound or mucous membrane with infectious material from a bat.

**Figure 4:**
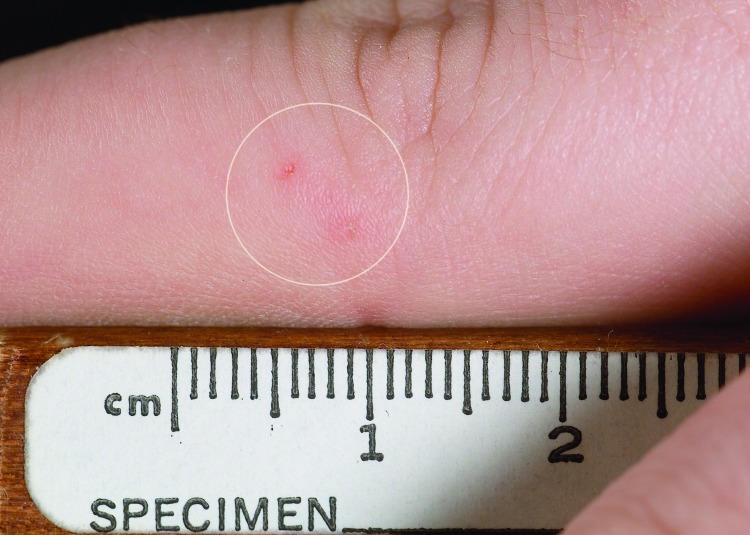
Wound inflicted by canine teeth of Eptesicus fuscus (big brown bat) while bat was being handled; picture taken same day as bite.

One hypothesis in the debate surrounding cryptic bat exposure and subsequent human rabies is that a bite from a bat is dismissed as insignificant or is unrecognized by the person because of somnolence or other impairment. For example, 32 human rabies cases have been caused by bat rabies virus variants from 1980 to 2004, but only 5 patients reported a bite from a bat. However, a bat "encounter" was recalled in 75% of cases, sometimes by family members or associates. Moreover, bat bites do not typically require medical attention for trauma from the bite itself (Figure 4).

Recognizing a potential exposure by the patient and appropriate administration of PEP by healthcare professionals is critical to maintaining the low rates of human rabies deaths observed in the United States. Although the rate is low, the number of human rabies cases caused by bat-associated rabies virus variants rose from 2 during the 1980s to 20 during the 1990s. This apparent increase in bat-associated human rabies cases led to changes in the recommendations for PEP to be considered in situations where a bat is physically present, a bite cannot be ruled out, and rabies cannot be ruled out by testing the bat. This cautionary language was formalized in the 1999 update of ACIP recommendations for human rabies prevention and control (*7*). Although these recommendations have been criticized (*11*), they have been widely implemented in public health practice. In addition to the ACIP recommendations, the public health response in New York consisted of updated rabies guidelines and an education campaign on bats and rabies during the late 1990s (*7**,**12**,**13*). Though these guidelines may have increased PEP, informed decision-making should always be used to reduce unnecessary PEP.

Despite increased educational emphasis on bats and rabies, public knowledge about the risk of rabies exposure from bats is lacking (*13**,**14*). A New York study documented that only 17%–26% of respondents knew that bats found in homes should not be immediately released (before considering the need to test the bat) (*13*). Additionally, a Colorado study found that at least a third of human encounters with bats that result in a possible exposure could have been prevented by adopting a "do not touch" approach to wildlife (*15*).

The deaths in 1993, 1994, and 1995 of 3 young girls in New York, Washington, and Connecticut and the death of a New Jersey man in 1997 (*16**–**19*) caused by a bat rabies virus variant elicited mass media attention. Sudden increases in PEP after highly publicized rabies cases or exposures have been previously described (*20**,**21*). Local and national events that involve a potential rabies case may affect how persons and physicians assess the risk of an animal exposure, perhaps leading to use of PEP in an environment of heightened concern rather than in response to a true exposure (*21**,**22*).

The 1999 ACIP guidelines (currently in the nascent stages of another update), as well as the availability of expert consultation at the local, state, and national level, should be widely promoted among healthcare professionals responsible for advising patients and providing PEP. Ultimately, public education about bats and rabies may increase the number of persons who seek PEP. A balanced approach is necessary to curtail inappropriate PEP and avoid unnecessary human deaths, such as the recent California case in which a patient did not seek PEP after a bat bite (*23*). Similarly, the recent Wisconsin human rabies case resulting from a bat bite was preventable had the risk been understood and had PEP been sought and appropriately administered. Survivorship in this case provides a welcome but extremely rare exception to the paradigm of rabies as inevitably lethal (*24*). It does not alter the ultimate goal of absolute human rabies prevention.
